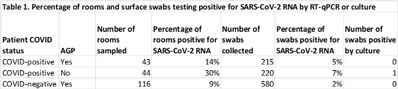# RNA and viable SARS-CoV-2 contamination of emergency department surfaces and association with patient COVID-19 status and aerosol procedures

**DOI:** 10.1017/ash.2022.203

**Published:** 2022-05-16

**Authors:** Windy Tanner, Scott Roberts, Douglas Barber, Elliana Barbell, Robert Heimer, Karen Jubanyik, Vivek Parwani, Jason Tanner, Andrew Ulrich, Martina Wade, Anne Wiley, Devyn Yolda-Carr, Richard Martinello

## Abstract

**Background:** Aerosol-generating procedures (AGPs) performed on COVID-19–positive patients raise concerns about the dissemination of SARS-CoV-2 via aerosols and droplets. Infectious aerosols and droplets generated by SARS-CoV-2–positive patient AGPs or through direct COVID-19 patient coughing or exhalation could potentially contaminate surfaces, leading to the indirect spread of SARS-CoV-2 via fomites within the emergency department (ED). We sampled surfaces of ED patient rooms occupied by known SARS-CoV-2–positive patients or patients under investigation for COVID-19 and undergoing an AGP to determine the frequency of room contamination with SARS-CoV-2 RNA. **Methods:** Swabs were collected from 5 room surfaces in the ED following AGPs performed on patients under investigation for COVID-19 or positive for SARS-CoV-2. High- and low-touch surfaces 6 feet (2 m) from the patient (door handle and return vent, respectively) and reusable medical equipment were swabbed. Swabs were tested for SARS-CoV-2 RNA by RT-qPCR; positive samples were cultured in Vero E6 cells. Patient COVID-19 results were confirmed through the electronic medical record. **Results:** In total, 203 rooms were sampled: 43 SARS-CoV-2–positive patients with an AGP, 44 SARS-CoV-2–positive patients who did not have an AGP, and 116 SARS-CoV-2–negative patients with an AGP, for a total of 1,015 swabs. Overall, SARS-CoV-2 RNA was detected on 36 (3.5%) surfaces from 29 rooms (14.3%) (Table 1). RNA contamination was detected more frequently in rooms occupied by SARS-CoV-2–positive patients who did not have an AGP than rooms occupied by COVID-19 patients (30% vs 14%). SARS-CoV-2 RNA was also detected in rooms occupied by SARS-CoV-2–negative patients undergoing an AGP (9%). SARS-CoV-2 RNA was most frequently detected on air vents (n = 15), bedrails (n = 10), equipment and vital signs monitors (n = 4 each), and door handles (n = 3). One bedrail was positive by culture and confirmed by an RT-qPCR cycle threshold reduction from >40 to 13. **Conclusions:** We detected SARS-CoV-2 RNA contamination on room surfaces in the ED, regardless of patient AGP or COVID-19 status; however, RNA contamination of room surfaces was most common in rooms occupied by SARS-CoV-2–positive patients who did not have an AGP, which may be attributable to stage of disease and viral shedding. SARS-CoV-2 RNA contamination was also present in rooms where APGs were performed on SARS-CoV-2–negative patients, suggesting carryover from previous patients. SARS-CoV-2 RNA was found most often on room air-return vents, further emphasizing the importance of aerosols in the spread of SARS-CoV-2.

**Funding:** None

**Disclosures:** None

**Table:**